# Determination of Scrub typhus Suggests a New Epidemic Focus in the Anhui Province of China

**DOI:** 10.1038/srep20737

**Published:** 2016-02-10

**Authors:** Min Cao, Li Che, Jinhai Zhang, Jianli Hu, Swaminath Srinivas, Ruiyao Xu, Henbing Guo, Yun Zhang, Changjun Wang, Youjun Feng

**Affiliations:** 1 Department of Disease Prevention and Control, Research Institute for Medicine of Nanjing Command, Nanjing, Jiangsu 210002, China; 2 Department of Medical Microbiology & Parasitology,Zhejiang University School of Medicine, Hangzhou, Zhejiang 310058, China; 3 Department of Biochemistry, University of Illinois at Urbana-Champaign, Urbana, IL 61801, United States of America; 4 Department of Acute Infectious Diseases Control and Prevention, Jiangsu Provincial Center for Disease Control and Prevention, China.

## Abstract

In 2007, 19 cases of a scrub typhus epidemic occurred within a week at a sports school in Mingguang County, Anhui Province, where no previous incidence of this mite borne disease had been reported. Sero-surveillance in 2009 indicated that 10 of the 100 school students possessed anti-*Orientia tsutsugamushi* antibodies. From 2009 to 2013, 60 small animals and 2250 mites were collected in the vicinity of the school. 5 of the Apodemus agrarius samples and 1 group of Leptotrombidium linhuaikongense tested positive via PCR for *O. tsutsugamushi*. Two strains of *O. tsutsugamushi* were identified by injecting Kun Ming (KM) mice peritoneally with the organs of either Apodemus agrarius or Leptotrombidium linhuaikongense. Apart from sharing 98% homology with the *O. tsutsugamushi* Yongworl strain, genes encoding the membrane protein from the two *O. tsutsugamushi* isolates shared >99% sequence homology with each other, reflecting the consistency of the pathogen in both the vector and the host. In addition, we also characterized a chronic scrub typhus infection in a local patient. The membrane protein gene fragment from the patient’s blood shared 99% homology with *O. tsutsugamushi Gillia*m strain, suggesting that more than one *O. tsutsugamushi* strain is present at this location.

Scrub typhus is a mite borne disease caused by the parasite *O. tsutsugamushi*, transmitted to humans and small animals by the bite of infected mites. Typical clinical features include sudden high fever, enlargement of lymph nodes, festering at the site of insect bite and rash, often accompanied by damage to the respiratory, digestive, nervous system and other organs, and in severe cases, organ failure or even death. China has a long history of scrub typhus epidemics. Since 1948, when *Orientia tsutsugamushi* was first identified in Guangzhou, China^1^, the outbreaks of scrub typhus have been frequently reported. Before 1985, the disease was most common during the summer and limited to southern China^2^,^3^,^4^,^5^. The most prevalent genotypes of *Orientia tsutsugamushi* reported in southern China were Karp, Gilliam, TA763 and TA716 types^6^. In fact the pathogenic strains responsible for “summer-type” scrub typhus were highly virulent and most likely transmitted by the *Leptotrombidium deliense* chigger. However, since 1986, the epidemic focus of scrub typhus has gradually moved northward. Outbreaks of scrub typhus occurring in the autumn and winter have been reported in Dongtai, Jiangsu Province since 1985, and thereafter in Henan, Shandong, Tianjin, Shanxi, Hebei, Jilin, Liaoning and Heilongjiang Provinces, which are all in northern China^7^,^8^,^9^,^10^. The main reservoir host of *O. tsutsugamushi* in these northern endemic areas is the wild mouse, *Apodemus agrarius*, while the predominant chigger vector is *L. scutellare* and the predominant genotypes of *O. tsutsugamushi* are Gilliam, Kawasaki and Yonchon^6^.

Scrub typhus has since been an emerging infectious disease in the Anhui Province of northern China. Though a single case was reported in 1982, in the county of Xiuning, in the southeast part of Anhui Province^11^, no further cases were reported over the next 25 years. In November 2007, a scrub typhus epidemic broke out at a sports school in Mingguang, a hilly area in the northeast of Anhui Province, with 19 students presenting with typical clinical symptoms of scrub typhus within a week of each other^12^. No cases of scrub typhus had previously been reported in this region suggesting a new epidemic focus. In 2008, another epidemic of scrub typhus occurred in Fuyang city, in the northwestern part of Anhui Province^13^. As an investigation into the natural foci of scrub typhus in this province hasn’t previously been conducted, this study was designed to investigate scrub typhus transmission in the vicinity of the sports school at which the epidemic of scrub typhus was reported in 2007. A focused investigation designed to identify *O. tsutsugamushi* genotypes, vector species and host animals present in Mingguang^13^ was conducted in order to highlight the features of scrub typhus at this location and to implement measures for preventing further dissemination of the disease.

## Results

### Serological survey

A scrub typhus epidemic consisting of 19 cases broke out in 2007 in a sports school in the Anhui province of northern China. A serological surveillance performed in 2009, using a colloidal gold reagent, indicated that 10 out of the 100 school students possessed anti-*O. tsutsugamushi* antibodies. On retrospection, five of the students who tested positive recalled that they had caught fevers or coughs in September 2008, the symptoms of which were alleviated after receiving oral doxycycline provided by a school doctor. The other 5 students recalled experiencing symptoms of a cold and received no treatment, suggesting the existence of sub-clinical infection at this location.

### Determination of host animals and vector mites

Small animals were collected annually from 2009 to 2013, in October using mouse cages positioned for a week in the vicinity of the sports school. In total 60 small animals were captured; 38 *Apodemus agrarius*, 16 *R. norvegicus*, 2 *Microtus fortis* and 4 *Neomys fodiens*, indicating that *Apodemus agrarius* was the dominant species, accounting for 63.3% of the total small animals captured (Table 1). 2250 mites were collected from the ears of the trapped small animals, consisting of 1980 *Leptotrombidium linhuaikongense* and 270 *Odontacarus majesticus*, indicating that *Leptotrombidium linhuaikongense* was the dominant mite species. The rate at which the small animals above carried mites are as follows: 60.5% (23/38) *Apodemus agrarius*, 31.3% (5/16) *R. norvegicus*, and 0 *Microtus fortis or Neomys fodiens*. The total rate of carrying mites was 46.7% (Table 1). Forty small animals including 25 *Apodemus agrarius*, 11 *R. norvegicus*, 2 *Microtus fortis and* 2 *Neomys fodiens* were captured in 2009. However, due to the rodent control strategies carried out by the sports school since 2010, annually, only 10, 5, 5 and 0 small animals were captured thereafter.

### Isolation of *O. tsutsugamushi*

**Figure 1:**
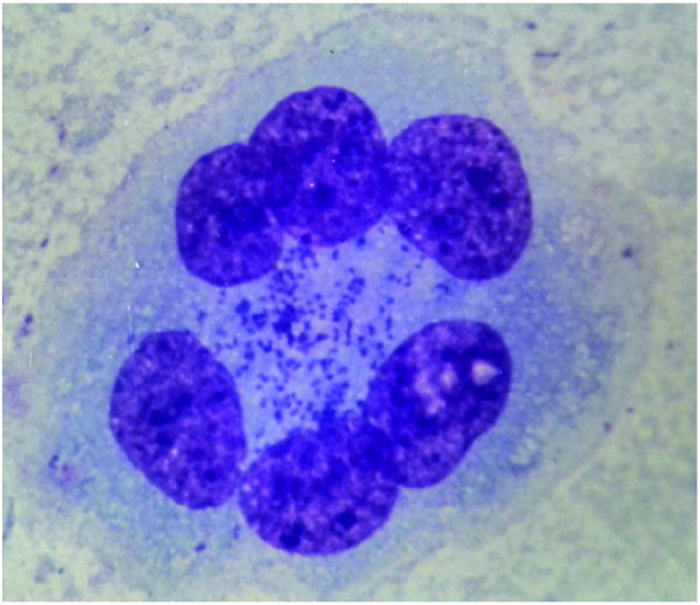
*Orientia tsutsugamushi* present in the liquid scraped from the abdominal wall of infected mice. The abdominal walls of infected mice were fixed and stained with Giemsa stain and presence of *O. tsutsugamushi* particles (purple particles) was assessed by light microscopy.

The 40 small animals trapped in 2009 were divided into 7 groups according to habitat and species; 3 groups of *Apodemus agrarius*, 2 groups of *Rattus norvegicus*, 1 group of *Microtus fortis* and 1 group of *Neomys fodiens*. The organs from these animals were dissected, triturated and pooled to yield one sample per group. The chiggers collected in 2009 and 2010 were pooled, then divided by species into one group of *Leptotrombidium linhuaikongense* and one group of *Odontacarus majesticus,* which were also triturated. KM mice were inoculated with these samples, and mice presenting symptoms of infection after two weeks were sacrificed. Liquid was scraped from the abdominal walls of infected mice, and the presence of *O. tsutsugamushi* particles was assessed by light microscopy. Mice inoculated by 1 specimen from *Apodemus agrarius* and 1 specimen of *Leptotrombidium linhuaikongense* presented typical features of *O. tsutsugamushi* infection, and this was confirmed via abdominal wall scrapings which revealed a *O. tsutsugamushi* infection (Fig. 1).

### PCR detection of *O. tsutsugamushi*

**Figure 2:**
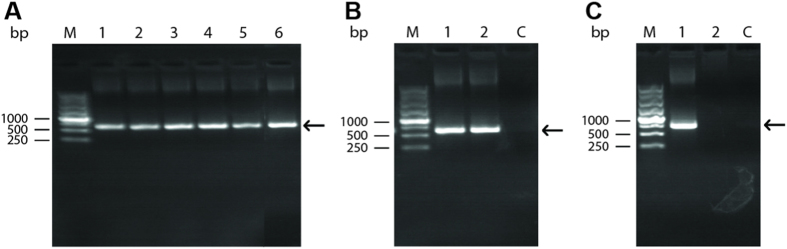
PCR-based assays for the presence of scrub typhus. (**A**) PCR products of the organs of wild small animals and chiggers M: 1 kb marker; 1–5: PCR products from the vicera of small animals; 6: PCR products from the chiggers. (**B**) PCR products from the organs of inoculated mice M: 1kb marker; 1–2: PCR products from the viscera of mice inoculated by the suspension of *Apodemus agrarius* (1) and *Leptotrombidium linhuaikongense* (*2*); C: health mice used as control. (**C**) PCR products from the patient with scrub typhus M: 1 kb marker; 1: PCR products from the blood of a patient with chronic infection in; 2: Negative amplification from the blood of the patient after symptomatic treatment; C: Blood from healthy adults used as control.

The organs of all 60 small animals and five groups of 20 chiggers (4 groups of *Leptotrombidium linhuaikongense* and 1 group of *Odontacarus majesticus*) and the organs of the infected mice, as described above, were triturated and DNA was extracted. *O. tsutsugamushi* infection was detected by PCR amplification of a specific 730 bp gene fragment of *O. tsutsugamushi*. The rate of detection of *O. tsutsugamushi* in wild small animals was 8.3% (Fig. 2A). The specific gene fragments of *O. tsutsugamushi* were also detected from the organs of inoculated mice (Fig. 2B)

When patient blood genomic DNA was used as a template, the 730 bp target gene was amplified by PCR and analyzed by agarose gel electrophoresis (Fig. 2C). However, after receiving doxycycline treatment for a month, the specific gene fragment of *O. tsutsugamushi* was absent from the patient’s blood (Fig. 2C).

### Sequence analysis and comparison

**Figure 3:**
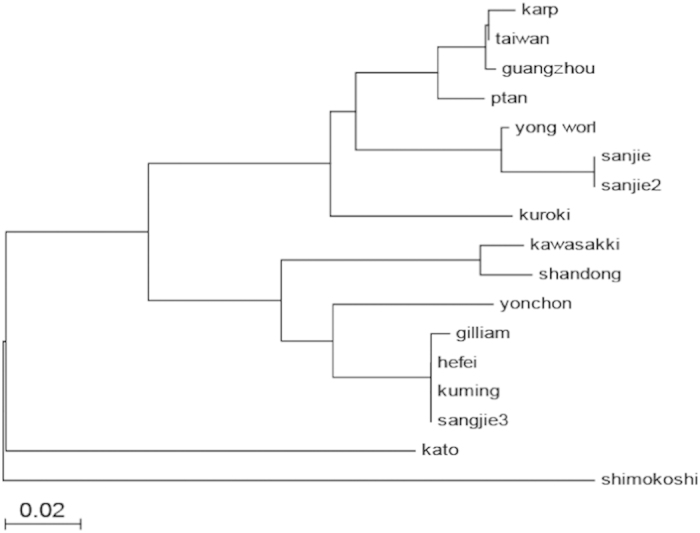
Phylogenetic tree of 16 *O. tsutsugamushi* strains constructed based on nucleotide sequence of 56 kDa protein. 16 *O. tsutsugamushi* strains including standard strains: *karp*, M33004; *Gilliam* M33267; *kato*, M63382; *Kuroki*, M63380; *Kawasaki* M63383 and *shimokoshi*, M63381; local strains from Korean: *yonchon* U19903; Yongworl, AF430141; local strains from China: *Ptan*, DQ288237; *Guangzhou*, AY283180; *Kunming*, GU446598; *Neimeng*, DQ514319; *Shandong*; DQ514320; *Taiwan*, GQ332758; *Hefei*, JX976614; *Sanjie*, KM095135; *Sanjie*2, KM115577; sanjie3, KM115578.

The 56 kDa protein sequences of the *O. tsutsugamushi* strains isolated from the *Apodemus agrarius* and *Leptotrombidium linhuaikongense* were designated sanjie and sanjie2, submitted to Genbank and assigned the serial number KM095135 and KM115577, respectively. The 56 kDa protein sequences of the *O. tsutsugamushi* isolates from the host animals and vector mites shared >99.8% homology, reflecting the consistency of pathogens between host and vector, and shared 98% homology with *O. tsutsugamushi* Yongworl strain (an isolate from Korea). Phylogenetic tree analysis (Fig. 3) also indicated that local strains and Korea young-worl strains belong to the same branch. The sequence of the specific gene fragment amplified from the patient’s blood was named sanjie3 and assigned the serial number KM115578. It shared 100% homology with *O. tsutsugamushi* Hefei strain and 99% homology to the *Gilliam* strain.

## Discussion

**Figure 4:**
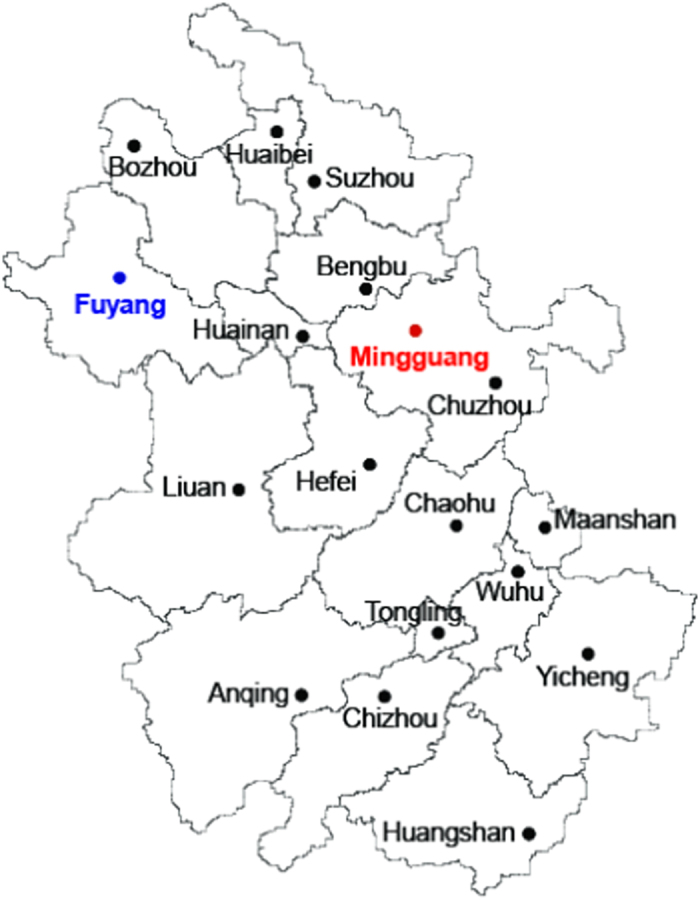
The endemic map for scrub typhus in Anhui province. The names of the cities of Mingguang and Fuyang are marked in red and blue respectively. The map of Anhui Province, China was created and processed using the software of Adobe Illustrator.

Since the appearance of scrub typhus in Mingguang, neither the epidemic foci of this infection nor the pathogen isolation in this area has been previously reported. An earlier report of another epidemic in Fuyang, a different area in the Anhui province (Fig. 4) merely mentioned the characteristic of the patients. This study conducts an epidemiological investigation of the natural focus of scrub typhus in the vicinity of a sports school that experienced an outbreak of scrub typhus in 2007. A 2009 sero-survey determined that 10% of students at this school possessed anti-*O. tsutsugamushi* antibodies. This rate is lower than that amongst the other residents of Mingguang, which was as high as 71.50% (143/200)^14^. This difference may be a reflection of the length of time spent by the patients in this area. Students of the sports school were mainly from different regions of China, while local residents were most likely to have resided in the area for their entire lives. In addition, students may have been less exposed than local residents to the vectors or vector hosts. Nonetheless, the high positive rate of anti-*O. tsutsugamushi* antibodies indicates a high risk of exposure to *O. tsutsugamushi* infection. Personal protection was recommended for those who frequently visit the native habitats of small animals and chiggers in the field.

The small wild animals trapped in autumn included *Apodemus agrarius, R. norvegicus, Microtus fortis* and *Neomys fodiens* with *Apodemus agrarius* accounting for 63.3% of the total small animals trapped. Although the number of the trapped small animals was small, likely due to the prevention strategies we recommended to reduce the risk of infection at this site, the pathogen was nonetheless detected in the dominant rodent species, *Apodemus agrarius,* indicating that it might be the major host for *O. tsutsugamushi* at the location, similar to reports from the neighboring provinces of Jiangsu and Shandong.

The two species of mites collected from the ears of trapped small animals were identified as *Leptotrombidium linhuaikongense* and *Odontacarus majesticus. Leptotrombidium linhuaikongense* was found in the Linhuaigang district of Huoshan County in Anhui province for the first time in 1959^15^, which is over two hundred miles away from the county of Mingguang. Although the specific gene fragment of *O. tsutsugamushi* had been amplified from this species of chigger previously^16^, we found no report that *Leptotrombidium linhuaikongense* is a vector of *O. tsutsugamushi*. In China several chiggers have been confirmed to be vectors of *O. tsutsugamushi*, including *Leptotrombidium deliense, Leptotrombidium rubellum, Leptotrombidium gaohuense, Leptotrombidium insulare, Jishou Leptotrombidium* and *Leptotrombidium scutellare*^17^. In this investigation, *Leptotrombidium linhuaikongense* was the dominant species in collected mites (88%). The specific gene segment of *O. tsutsugamushi* was amplified and a new *O. tsutsugamushi* strain was isolated, indicating that this species of mite is likely to be a new vector of scrub typhus. A specific gene fragment of *O. tsutsugamushi* was also amplified from the blood of a patient with a chronic *O. tsutsugamushi* infection, residing near the sports school. Based on the patient’s account, we can infer that patient remained infected for about 9 months before symptomatic treatment. After correct diagnosis of scrub typhus, he took doxycycline 0.2 g a day for a month, and the symptoms were alleviated and eventually resolved. PCR detection for a specific gene fragment of *O. tsutsugamushi* in the patient’s blood confirmed that the infection was then cleared. This is the first report of a chronic scrub typhus infection in China.

In fact, chronic infection of *O. tsutsugamushi* is a common phenomenon among animals, with some kinds of animals carrying the pathogen for a long period without any symptoms^18^,^19^. Persistence of the pathogen in the human body is rarely reported, mostly because medical staff rarely track the persistence of pathogens after resolution of clinical symptoms. Moon-Hyun Chung and others issued a detailed report in 2013^20^ where they found that a specific DNA fragment could still be detected in the patient’s blood four months after infection. *O. tsutsugamushi* was isolated from the blood of a patient 18 months after infection following symptomatic treatment, and in the absence of typical clinical symptoms of scrub typhus. In our study, the patient had never received the symptomatic treatment before correct diagnosis, which may explain the detection of *O. tsutsugamushi* DNA in the blood nine months after infection. The anti-*O. tsutsugamushi* antibodies in this patient’s serum could not be detected by the colloidal gold reagent developed by our group, while a more sensitive ELISA determined the anti-*O. tsutsugamushi* IgG antibody titer of the patient’s serum to be 1:400. This result echoes a previous report that the anti-*O. tsutsugamushi* antibody titer in the serum of patients would be significantly reduced 4 to 8 months after infection, in comparison with that during the acute period^18^. In this case disease was often reported in October or November, in accordance with cases reported north of the Yangtze River in China, which all occurred in autumn or winter. The Gilliam and Karp genotypes of *O. tsutsugamushi* are thought to be most prevelet in south of Yangtze River^21^, while Kawasaki and Gilliam types were mainly found in north of the Yangtze River^22^,^23^. In this study, sequences of the gene encoding the 56 kDa outer membrane protein of the two *O. tsutsugamushi* isolates from wild small animals and vector mites shared >99.8% homology, reflecting the consistency of pathogens between host and vector. They also shared 99% homology with *O. tsutsugamushi* Yongworl strain, an isolate from Korea which had previously never been found elsewhere in China. Phylogenetic tree analysis also indicated that the local strains and Korea young-worl strains belong to the same branch. Meanwhile, the sequence of the specific gene fragment of *O. tsutsugamushi* from the patient’s blood shared 100% homology with that from the first patient in the city of Hefei, and is closely related to the Gilliam strain.

These results suggest that more than one strain of *O. tsutsugamushi* is circulating in this area. Our conclusions are limited by the number of blood samples we could obtain from local patients diagnosed with scrub typhus. During the outbreak of scrub typhus in 2007, only patients’ sera were retained, while the clot was discarded due to lack of prior experience. Hence the genotype of *O. tsutsugamushi* could not be determined at that time. Although cases of scrub typhus have been occasionally reported in recent years, patient specimens were not obtained in a timely fashion as it was not required by law to officially report scrub typhus in China. In addition, the disease is often misdiagnosed due to sharing similar symptoms with other febrile diseases such as the common cold. We cannot determine the prevalence of *O. tsutsugamushi* strains at this site through analysis of only one patient. Thus, further work will be required to continue this line of research.

In conclusions, this is the first report identifying vectors and animal hosts of the *O. tsutsugamushi* genotypes in Mingguang. Briefly, *Apodemus agrarius* and *Leptotrombidium linhuaikongense* are likely to be the most prevalent host and vector of scrub typhus in this area. Gilliam and Yongworl-like strains of *O. tsutsugamushi* were found. Chronic scrub typhus infection was also reported for the first time in a Chinese patient. These studies highlight the features of scrub typhus at the location and may provide useful information for further understanding the distribution of scrub typhus in China, facilitating targeted prevention and treatment of the disease.

## Methods

### Ethics Statement

The study was conducted on private lands, in the sports school and in the wild field. Prior permission was obtained from the president of the sports school, Mr. Huadong Huang, and the owners of the private lands named Mingqiang Li and Xing Xu. In China, no ethical approval is needed for sampling rodents outside protected areas and the field study didn’t involve any endangered or protected species.

All animal experiments were approved by the Ethics Committee of the Research Institute for Medicine, Nanjing Command. These methods were carried out in accordance with the approved guidelines. Human participation in this study was approved by the Ethics Committee of Research Institute for Medicine of Nanjing Command and the people provided written informed consent.

### Blood sample collection for serological surveys

In 2009, 100 sera samples of students, over the age of 18, attending the sports school at which scrub typhus was detected in 2007 were collected. The *O. tsutsugamushi* specific antibody content of participant sera was detected using colloidal gold diagnostic reagents, as previously described^24^.

### Blood sample collection from a patient chronically infected with *O. tsutsugamushi*

In May 2013, a young male presented with enlargement of neck, armpit and inguinal lymph nodes of unknown etiology. The patient described in retrospection that he had a fever over 39 °C in September 2012 which was accompanied by a rash all over the body. The fever persisted for 2 weeks despite administration of cephalosporin antibiotics, then resolved. He recalled that he was bitten by an insect which left an eschar-like scar near his navel. However, the doctor ignored this feature at that time. This patient had subsequently experienced repeated mild fever accompanied by lymph node enlargement, and had sought medical attention, but was not properly diagnosed. As this patient was resident in the vicinity of the school at which the 2007 scrub typhus epidemic was detected, his blood sample was eventually sent to our laboratory for analysis.

### Field Sample collection

Small animals were collected in October, annually, from 2009 to 2013. Mouse cages were used to capture mice for a week in locations including houses, vegetable fields, peanut fields, grasslands, ridges and other areas where small animals such as rodents were often seen around or in the sports school. The cages were placed at nightfall and retrieved at dawn and then the trapped rodent species were identified and the dominant species was determined. The mites present inside the ears of each rodent were collected, counted, and the species were identified by microscopy and the dominant mite specie was determined. The small animals were anesthetized by ether and the organs, including the livers, spleens and kidneys, were dissected under sterile conditions and reserved for PCR detection and pathogen isolation. The remains of each rodent specimen were stored at −80 °C. The mite specimens were used for PCR detection and pathogen isolation or stored in 70% ethanol.

### Isolation of *O. tsutsugamushi*

The 40 small animals trapped in 2009 were divided into 7 groups according to habitat and species; 3 groups of *Apodemus agrarius*, 2 groups of *Rattus norvegicus*, 1 group of *Microtus fortis* and 1 group of *Neomys fodiens* with 2~3 small animals per group. The organs of these animals were acquired as previously described, triturated and suspended in SPG sucrose phosphate buffer to a final concentration of 10%, and pooled to yield one sample per group. The chiggers collected in 2009 and 2010 were pooled, then divided by species into one group of 20 *Leptotrombidium linhuaikongense* and one group of 20 *Odontacarus majesticus,* which were also triturated. 10 mice of the Kunming (KM) species for each group (purchased from Academy of Military Medical Science) weighing 15 to 18 g each were intra-peritoneally injected with 0.5 ml of one sample. The inoculated mice were observed carefully twice daily. Two weeks after inoculation, mice presenting symptoms such as weakness, trembling and erect hair, were sacrificed after about two weeks. Liquid was scraped from the abdominal walls of infected mice, then fixed and stained with Giemsa stain, and the presence of *O. tsutsugamushi* particles was assessed by light microscopy.

### PCR detection of *O. tsutsugamushi* from field trapped small animals and mites, the inoculated mice and patient’s blood

The organs (including liver, spleen and kidney) of 60 small animals and five groups of 20 chiggers (4 groups of *Leptotrombidium linhuaikongense* and 1 group of *Odontacarus majesticus*) were triturated and DNA was extracted using the Invitrogen DNeasy Blood & Tissue kit, following the manufacturer’s instructions. DNA were extracted from organs of the inoculated mice presenting obvious infection of *O. tsutsugamushi* and from the patient’s blood using the same Invitrogen kits. PCR was performed with the following primers designed according to the gene sequence of the 56 kDa outer membrane protein of *O. tsutsugamushi* standard strains; P1: 5′-gagcagagataggtgttatgtacc-3′, P2: 5′-tatacccatcaaaaagatctctgagc-3′^25^ and synthesized by Sangon Co. (Shanghai, China). The PCR reaction had a final concentration of 200 μmol/L of each of deoxynucleoside triphosphate, 0.3 μmol/L of each primer, and 0.6 units of Taq polymerase in 10 mmol/L Tris-HCl working buffer containing 2.0 mmol/L MgCl_2_ and 50 mmol/L KCl, pH 8.3, provided by TaKaRa Co., Dalian, China. The PCR conditions are as follows: 5 min at 95 °C, followed by 35 cycles of 60 s at 94 °C, 60 s at 56 °C, and 60 s at 72 °C. The reaction ended with a final step of 7 min at 72 °C. PCR products were analyzed on 1% agarose gel, and the sizes of the PCR products were determined against a standard molecular weight marker (Fermentas MBI Co., Lithuania, and TaKaRa Co.).

### Sequencing and Analysis

The amplified gene fragments were sequenced by TAKARA Co. and the sequences were submitted to Genbank. The sequences were subjected to BLAST and edited by Bioedit, then, aligned and compared with those of several other *O. tsutsugamushi* strains by Clustal X. Finally phylogenetic analysis was performed with Mega 4.0, utilizing the minimal evolution algorithm (minimal evolution, ME).

## Additional Information

**How to cite this article**: Cao, M. *et al*. Determination of Scrub typhus Suggests a New Epidemic Focus in the Anhui Province of China. *Sci. Rep.* **6**, 20737; doi: 10.1038/srep20737 (2016).

## Figures and Tables

**Table 1:** Host animals and vector mites of *O. tsutsugamushi* disease in Mingguang area.

Rodent species	Apodemus agrarius	*Rattus norvegicus*	Microtus fortis	Neomys fodiens	Total
Number of trapped animals	38 (62.5%)	16 (26.6%)	2 (3.3%)	4 (6.7%)	60
Trapped animals carrying mites	23 (60.5%)	5 (33.3%)	0 (0%)	0 (0%)	46.7%
